# Accuracy and Early Outcomes of Patient-Specific TKA Using Inertial-Based Cutting Guides: A Pilot Study

**DOI:** 10.3390/medicina61091554

**Published:** 2025-08-29

**Authors:** Gianluca Piovan, Andrea Amarossi, Luca Bertolino, Elena Bardi, Alberto Favaro, Lorenzo Povegliano, Daniele Screpis, Francesco Iacono, Tommaso Bonanzinga

**Affiliations:** 1 IRCCS Ospedale Sacro Cuore Don Calabria, Viale Luigi Rizzardi 4, Negrar di Valpolicella, 37024 Verona, Italy; piovan.gianluca@hotmail.it (G.P.); andrea.amarossi@gmail.com (A.A.); lorenzo.povegliano@sacrocuore.it (L.P.); daniele.screpis@sacrocuore.it (D.S.); 2 Department of Biomedical Sciences, Humanitas University, Via Rita Levi Montalcini 4, Pieve Emanuele, 20072 Milan, Italy; luca.bertolino@st.hunimed.eu (L.B.); tommaso.bonanzinga@hunimed.eu (T.B.); 3 IRCCS Humanitas Research Hospital, Via Manzoni 56, Rozzano, 20089 Milan, Italy; elena.bardi@humanitas.it (E.B.); francesco.iacono.bo@gmail.com (F.I.)

**Keywords:** arthroplasty, replacement, knee, osteoarthritis, knee, knee prosthesis, patient-specific modeling, patient-specific instrumentation, patient-specific implant, additive manufacturing, navigation system, accelerometer-based navigation

## Abstract

*Background and objectives*: Patient-specific components (PSC) represent an innovative option for total knee arthroplasty (TKA) in advanced osteoarthritis. Their effectiveness, however, closely relies on accurate positioning. Our study investigates the accuracy achieved by means of an inertial-based extramedullary cutting guide and the postoperative clinical and radiographic outcomes. *Methods and materials*: This was a prospective, single-arm, pilot study involving patients undergoing primary TKA with YourKnee^TM^ PSC. Femoral and tibial bone resections were performed using the Perseus inertial-based extramedullary cutting guide. Postoperative mechanical alignment and component positioning were assessed by computed tomography. Clinical outcomes were evaluated preoperatively and at 1, 3, 6, and 12 months postoperatively by main knee function and clinical outcome measures. *Results*: The study population included a small cohort (*n*= 12, four females/eight males, mean age 69 ± 5.65 years, mean BMI 25.7 ± 3.8 kg/m^2^, KL grade > 3) with no control group. The mean absolute error between the planned and obtained Hip–Knee–Ankle angle was 1.36° ± 1.06 and within ±3° of all cases. Mean coronal alignment error was 1.87° ± 0.87 and 1.67° ± 0.75 for the femoral and tibial components, respectively. The mean sagittal alignment error was 1.89° ± 1.24 and 2.45° ± 0.87 for the femoral and the tibial components, respectively. Patients showed significant improvement in clinical and functional scores within the first 6 months: OKS increased from 20.64 ± 2.77 at the preoperative screening to 42.27 ± 4.34 (*p* < 0.0001), total KSS rose from 90.64 ± 17.25 to 169.36 ± 23.57 (*p* < 0.0001), and FJS reached 85.09 ± 17.14 at 6 months (*p* = 0.0031), indicating excellent functional recovery and forgotten joint effect. Knee ROM improved from 90.91° ± 11.14 to 110.36° ± 8.44 (*p* < 0.0001). After 6 months, outcome scores plateaued, suggesting an early stabilization of clinical benefits. No signs of radiolucency were detected on X-rays at 3- and 12-month follow-ups. *Conclusions*: The Perseus inertial-based extramedullary cutting guide used in combination with the YourKnee^TM^ PSCs resulted in accurate intraoperative prosthesis positioning and significant improvements in clinical and functional outcomes at 6 months after surgery. Despite the small sample size and absence of a control group, the results suggest that such combination represents a viable option to conventional surgical instrumentation and current off-the-shelf prosthetic designs.

## 1. Introduction

To date, total knee arthroplasty (TKA) remains the gold standard treatment for patients with advanced knee osteoarthritis (OA). Nonetheless, data from patient reported outcome measures (PROMs) highlight that 7 to 20% of patients remain dissatisfied after surgery—most commonly due to residual knee pain and suboptimal knee functional status, including restricted range of motion (ROM) [1].

These shortcomings are partly attributed to the inherent limitations of current prosthesis designs and limited sizing options [2] Off-the-shelf (OTS) knee prostheses often fail to accommodate the wide anthropometric differences among populations, ethnicities, and genders [3]. As a result, a considerable percentage of patients received sub-optimal implant sizes [2,4,5], undermining the restoration of both tibio-femoral and patello-femoral kinematics.

In recent years, preoperative planning in orthopedic surgery has significantly evolved with the integration of advanced imaging modalities and additive manufacturing technologies. Computed tomography (CT)-based 3D reconstruction provides accurate anatomical details which, together with the use of advanced additive manufacturing processes, serve as the foundation for designing patient-specific instruments (PSI) and patient-specific prosthetic components (PSCs) [6]. PSCs seek to minimize surgical compromises by tailoring implant design to the patient’s unique anatomy, thereby aiming to restore native joint kinematics [7]. By closely replicating their pre-arthritic anatomical condition, PSCs offer a more individualized approach to joint reconstruction [8]. Paradoxically, such personalization also facilitates standardization of the surgical technique, as it minimizes the need for intra-operative adaptations [8].

The effectiveness of knee PSCs, however, closely depends on the precision and replicability with which they are positioned in relation to preoperative planning. In recent years, increasing efforts have been made to develop advanced surgical systems to enhance prosthesis positioning, including navigation and robotic systems. While navigation-assisted and robotics-assisted TKA have demonstrated superior component positioning accuracy, these benefits come with the trade-off of a steep learning curve, need for additional technical support, and overall increased surgical time [9,10]. As a more user-friendly and cost-effective alternative, inertial-based cutting guides have demonstrated good intra and inter-operator reliability in assisting the surgeon during tibial and femoral bone cuts, and superior performance in restoring the mechanical axis compared to conventional TKA [11,12,13,14,15].

So far, the positioning accuracy of knee PSCs using inertial-based cutting guides and their associated clinical outcomes have never been reported.

The aim of this study was to assess the accuracy of an inertial-based extramedullary Perseus cutting guide (Orthokey, Florence, Italy) in the positioning of knee PSCs in patients surgically treated for primary OA and to evaluate the clinical and radiographic outcomes resulting from the combination of PSCs implants and this advanced positioning technology.

## 2. Materials and Methods

### 2.1. Study Design and Setting

This was a single-center prospective case series performed at IRCCS Sacro Cuore Don Calabria (Negrar di Valpolicella, Verona, Italy) between May 2022 and October 2022. The study had been approved by the Ethical Committee of Verona—Rovigo (Italy) (Protocol code: 2020-ZA, Protocol version: 1.7, date: 30 April 2020) and was conducted in agreement with the Declaration of Helsinki and hospital guidelines for Good Clinical Practice. Informed consent had been obtained from all individual participants included in the study.

### 2.2. Patients

The study enrolled patients with primary OA undergoing TKA. Inclusion criteria were knee OA Kellegren–Lawrence (KL) grade ≥ 3, age between 55 and 80 years, axial deformity < 15°. Exclusion criteria were an American Society of Anesthesiologists (ASA) score ≥ 3, a previous contralateral TKA, revision surgery of a previous TKA, lower limb fracture sequelae, severe tibial and femoral bone defect, previous patellectomy, femoral or tibial osteotomy outcomes, or body mass index (BMI) ≥ 40.

Patient data collected included demographic information: age at time of surgery, operated side, height, weight, BMI, KL OA grade, and ASA score.

### 2.3. CT Evaluation: Preoperative Planning and Postoperative Measurements

Computed Tomography (CT) scans of the lower limb were performed four weeks prior to and one month immediately after surgery using the same instrument (SOMATOM Definition Flash, Siemens Healthcare, Erlangen, Bayern, Germany) with the following settings: maximum slice thickness of 0.8 mm, resolution of 512 × 512 pixels, minimum of 120 kVp.

The preoperative CT scan was used to plan bone cuts at the distal femur and proximal tibia and to determine the appropriate sizing of the PSCs, as further detailed in the Section 2.4. The postoperative CT scan was used to evaluate the positioning of the PSCs after implantation and the overall limb alignment by comparing it with the planned one, as detailed in Section 2.5.

In the coronal plane, the Medial Distal Femoral Angle (MDFA) and the Medial Proximal Tibial Angle (MPTA) were considered and used to compute the CT-derived Hip-Knee-Ankle ($HKA_{CT}$) angle as:

$$HKA_{CT}=MDFA+MPTA\;-\;180{}^{\circ}$$

**Figure 1:**
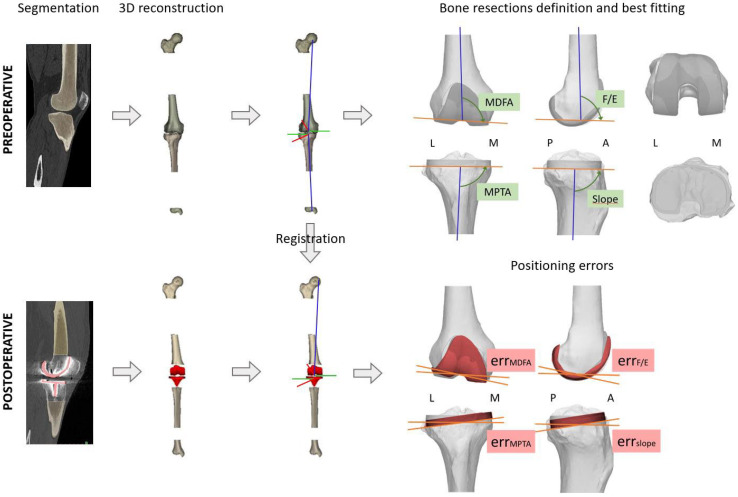
Full CT Workflow from preoperative planning to postoperative component positioning evaluation (L = lateral, M = medial, A = anterior, P = posterior, err = difference between the pre- and post-operative positioning).

Varus angles were reported as negative values, whereas valgus angles were reported as positive values. In the sagittal plane, the tibial slope and the femur Flexion–Extension (F/E) angle—computed relative to the tibial and femoral mechanical axes, respectively—were measured. A tibial slope greater than 90° indicates posterior tilting, while an F/E angle greater than 90° indicates anterior tilting (flexion). The CT workflow is reported in Figure 1.

### 2.4. Preoperative Planning

CT scan data were imported as DICOM images into Materialise Mimics 23.0 (Leuven, Belgium) for bone segmentation. Grayscale thresholding was applied to distinguish the bones from the surrounding soft tissues based on their density. Bone models were loaded into Materialise 3-matic 15.0 (Leuven, Belgium) for 3D data visualization and processing.

The mechanical axes and reference systems of the femur and tibia were identified by connecting the center of the femoral head to the intercondylar notch for the femur and the center of the tibial spine to the center of the ankle joint for the tibia [16]. Coronal femoral resection was planned perpendicular to the trochlear groove bisector, according to the principle described by Iacono et al. [17]. Tibial resection was performed to match the thickness of the tibial component, with adjustments for cartilage wear, in order to restore the native pre-arthritic joint line and the patient’s knee geometry, while ensuring that the $HKA_{CT}$ angle remained within ±3° from neutral.

In all the patients, a cruciate retaining (CR) PSC (YourKnee^TM^ CR System by Rejoint S.r.l., Castel Maggiore, Bologna, Italy) was implanted. Each YourKnee^TM^ PSC was designed considering the anatomy reconstructed from the preoperatory CT scan and the target femoral and tibial resection angles, so as to maximize the bone coverage and minimize underhang and overhang. The selection of the best-fitting prosthetic components was carried out with the company’s internally developed and validated software using a process that matched the femur and tibia 3D-reconstructed anatomy with over 9000 prosthetic solutions. Specifically, the femoral component was selected considering combinations of different antero-posterior (AP) and medio-Lateral (ML) dimensions. The height of the posterior condyles and the trochlear shape could be also tunable according to the patient’s specific anatomy. Tibial components were selected similarly by evaluating combinations of different AP and ML dimensions until the best-fitting one is identified.

As for sagittal resections, femoral flexion was defined to prevent anterior notching, while the tibial slope was set to match the anatomical slope of the medial side of the plateau.

Following surgeon preoperative planning and design approval, YourKnee^TM^ PSCs were manufactured through a 3D-printing additive process.

### 2.5. Postoperative Measurements

Postoperative CT images of the operated knee were loaded into Materialise Mimics 23.0 (Leuven, Belgium) and 3D models of the femur and tibia were generated. Differently from the preoperative phase, bone sections around the metallic components were removed from the bone segmentation, as they were affected by artifacts (e.g., scattering effect) linked to the presence of the prosthesis metallic components. For the same reason, prosthesis components could not be directly segmented from the postoperative CT images. Instead, the spatial position and orientation of the prosthesis components were identified by the manual superimposition of their computer-aided designed (CAD) 3D models onto the CT images.

On the preoperative and postoperative 3D bone reconstructions, distinct anatomical landmarks visible in both scans were identified. These included the apex of the lesser and greater trochanters, the center of the femoral head (determined via spherical fitting), and subject-specific features such as details of the linea aspera for the femur, the medial malleolus, the center of the ankle joint, the tibial tuberosity, and additional features along the soleal line. These landmarks were used to superimpose (register) the preoperative and postoperative 3D models through a rigid 6-degree-of-freedom transformation. After registering the preoperative and postoperative reconstructions, the deviation between the preoperative planned and the postoperative actual components’ positioning was measured in terms of MDFA, MPTA, $HKA_{CT}$ angle, tibial slope, and femur F/E.

### 2.6. Clinical, Functional, and Radiographic Evaluation

Patients were clinically evaluated four weeks prior to surgery using the Oxford Knee Score (OKS) and Knee Society Score (KSS) clinical questionnaires, and the measurement of joint ROM using a goniometer. Clinical follow-up was performed at 1, 3, 6, and 12 months after surgery and included the acquisition of the same indexes (OKS, KSS, ROM), as well as the Forgotten Joint Score (FJS). Additionally, full leg X-rays radiographs were obtained at 3- and 12-months follow-ups evaluated to detect the presence and location of radiolucency lines according to the Knee Society Total Knee Arthroplasty Roentgenographic assessment [18].

### 2.7. Surgical Procedure

All surgical procedures were performed by an experienced surgeon (G.P). The surgical approach was performed via medial parapatellar skin incision and a subvastus capsular approach.

**Figure 2:**
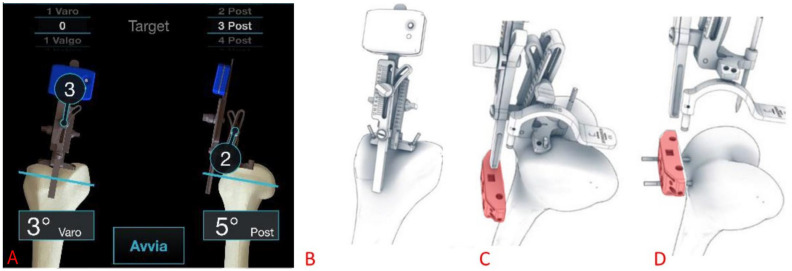
(**A**) Intraoperative screenshot of the Perseus interface for the definition of the femoral distal cut. Above, the target coronal and sagittal slopes. On the bottom, the current position of the Perseus. In the circles, the correction to provide to achieve the target values. (**B**) The system is locked in place with two additional pins fixed on the trochlear shoulders. (**C**) The cutting block (in light red) is inserted along the Perseus longitudinal bar so as to replicate the desired coronal slope. (**D**) The cutting block is fixed in position and the Perseus system is removed.

The distal femoral cuts were performed using the Perseus inertial-based extramedullary smart cutting guide as shown in Figure 2 (Figure 2A,B) [12]. The knee was in flexion position and a pin was inserted into the center of the femur at the most distal point of the trochlear groove (intercondylar notch). The system was secured with two additional pins fixed on the trochlea.

**Figure 3:**
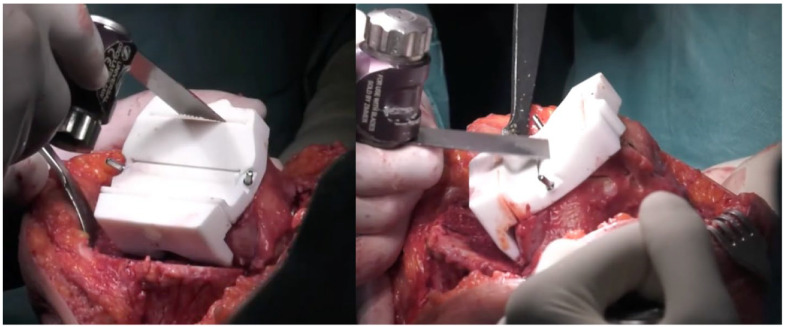
Femoral “4-in-1” 3D-printed patient-specific PSI guide with posterior reference.

After reaching the desired resection angle according to the preoperative planning, the distal femoral resection was carried out with the aid of a bespoke femoral cutting block designed to be locked in place according to the resection angle set by the Perseus system (Figure 2C,D). Additional femoral resections were then performed using a patented “4-in-1” 3D-printed patient-specific guide with a posterior reference that adheres on the posterior condyles (Figure 3).

**Figure 4:**
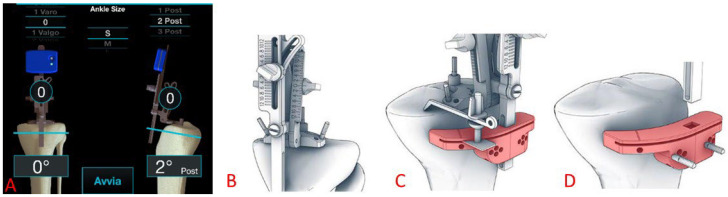
(**A**) Intraoperative screenshot of the Perseus interface for the definition of the tibial proximal cut. Above, the target coronal and sagittal slopes. On the bottom, the current position of the Perseus. In the circles, the correction to provide to achieve the target values. (**B**) The system is locked in place with two additional pins fixed on the tibial plateau. (**C**) The cutting block (in light red) is inserted along the Perseus longitudinal bar so as to replicate the desired coronal slope. (**D**) The cutting block is fixed in place and the Perseus system removed.

The tibial cut (Figure 4) was performed using the Perseus system, placing a central pin on the tibial spine, and two additional pins on the plateau for proper cutting guide stabilization. After the system was set to the desired coronal resection angle, a tibial resection block was fixed and the bone was resected.

After completing bone resections, knee stability was assessed using trial components, performing a varus–valgus stress at different ROM degrees. Subsequently, the final prosthetic components were implanted using PALACOS^®^ R cement (Heraeus Medical GmbH, Philipp-Reis-Straße 8/13, 61273 Wehrheim, Germany). Patellar replacement was not performed in any case, but cheilectomy and peripheral neurotomy were carried out.

The mean surgical time was 78.9 ± 12.5 min (range: 61–98 min), and the mean intraoperative blood loss was 262.5 ± 88.2 mL (range: 200–500 mL). No intraoperative complications were observed.

### 2.8. Statistical Analysis

Radiological outcomes were evaluated based on the mean and standard deviation of the absolute differences between preoperative planning and postoperative measurements. Agreement between the planned and measured values was further assessed using a Bland–Altman analysis and paired *t*-tests in GraphPad Prism 10.4.1 (GraphPad Software, Boston, MA, USA), with a significance level set at *p* < 0.05.

Clinical outcomes across all time points were analyzed using repeated measures ANOVA in GraphPad Prism 10.4.1 (GraphPad Software, Boston, MA, USA), with a significance level set at *p* < 0.05. Sphericity was not assumed, and Greenhouse–Geisser corrections were applied. The normality of residuals was assessed using quantile–quantile (QQ) plots. When a significant effect was observed, post-hoc pairwise comparisons were performed using Bonferroni correction to assess differences between all the time points: pre-operative, and post-operative at 1, 3, 6, and 12 months.

## 3. Results

### 3.1. Population Demographics

The study included twelve patients (four females, eight males), with a mean age of 69 ± 5.65 years and a mean BMI of 25.7 ± 3.8 kg/m^2^. Five patients had KL grade 4 OA, and seven had grade 3. Ten patients were classified as ASA score 2, and two as ASA score 3. Six procedures were performed on the right knee and six on the left. In one patient, a software issue required the use of conventional instrumentation to perform the proximal tibial bone cut, leading to the exclusion of this patient from the study. This reduced the cohort available for data analysis to *n* = 11.

Although the study was designed as an exploratory pilot investigation, a posteriori power analysis based on literature-derived assumptions provided a theoretical validation of the enrolled cohort. The coronal alignment of the femoral and tibial components was identified as the primary endpoint for power estimation. A clinically significant difference of 3° was considered, with a standard deviation of 2° assumed based on previous similar studies [19]. Under these assumptions, using a significance level (alpha) of 0.05, the statistical power of the paired *t*-test with 11 participants was calculated to be greater than 99%.

### 3.2. Positioning Accuracy Achieved with Inertial-Based Extramedullary Cutting Guides

**Figure 5:**
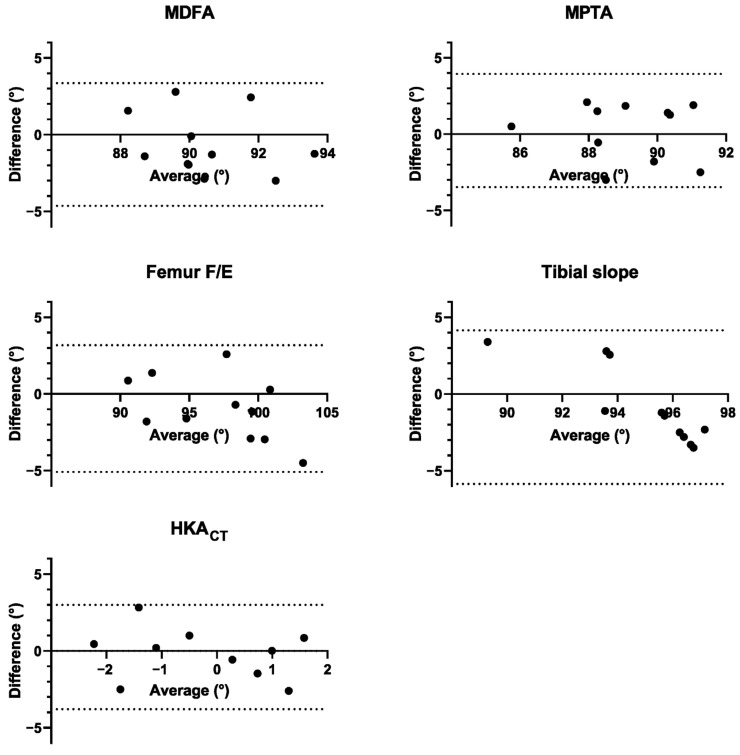
Bland–Altman plots comparing planned and post-operative alignment angles. Each plot shows the difference (bias) between planned and actual measurements, with dotted lines indicating the 95% limits of agreement (±1.96 SD).

Table 1 reports radiological outcomes, including planned and post-operative mean angles, mean absolute errors, percentage of cases with an absolute error below the clinically-relevant level of 3°, Bland–Altman bias, and 95% limits of agreement (LoA). The bias represents the average difference between planned and post-operative values, while the LoA represents the range within which 95% of these differences lie. Corresponding Bland–Altman plots are shown in Figure 5. Overall, the results indicate good agreement between planned and post-operative values. In the coronal plane (i.e., MDFA, MPTA), the absolute error fell below the 3° threshold in all the cases and the Bland–Altman analysis indicated minimal systematic bias and narrow LoA. In the sagittal plane (i.e., Femur F/E and Tibial slope), a few cases exceeded the 3° threshold, with higher biases and wider LoA observed. For $HKA_{CT}$, absolute errors were consistently below 3°, with minimal bias and narrow LoA. Additionally, no statistically significant differences were found between planned and postoperative values.

### 3.3. Postoperative Clinical and Radiographic Outcomes

Clinical and functional mean outcomes, along with the overall *p*-values, are reported in Table 2 for each time point. QQ-plots of the residuals are reported in Figure S1 of the Supplementary Materials.

**Figure 6:**
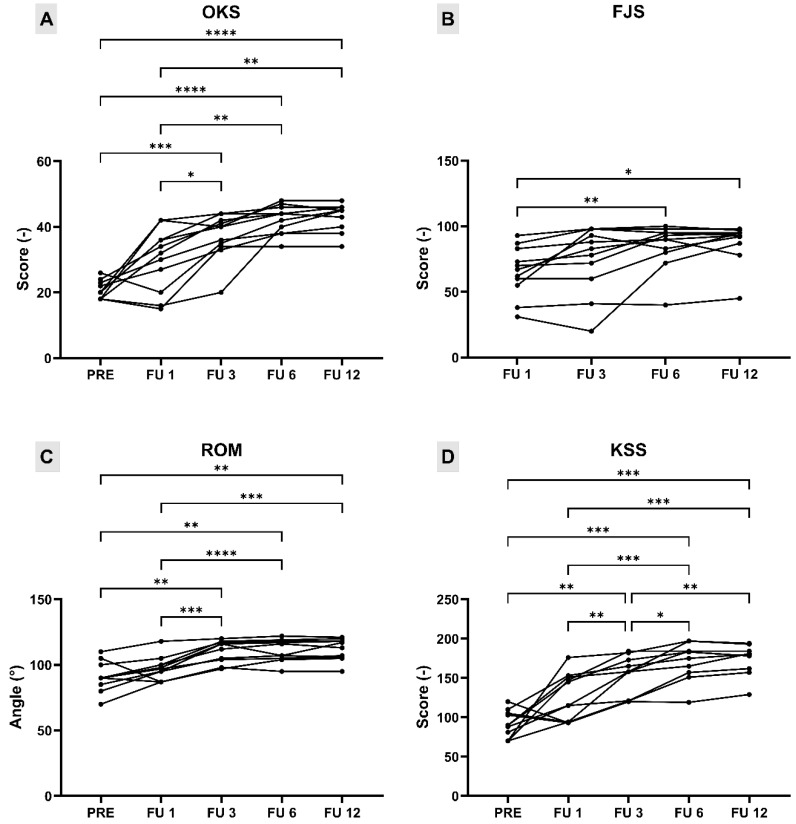
Pairwise comparisons of (**A**) Oxford Knee Score (OKS), (**B**) Forgotten Joint Score (FJS), (**C**) knee range of motion (ROM), and (**D**) Knee Society Score (KSS) at pre-operative (PRE) baseline and at 1-, 3-, 6-, and 12-month follow-ups (FU). Significance levels: * *p* ≤ 0.05; ** *p* ≤ 0.01; *** *p* ≤ 0.001; **** *p* ≤ 0.0001.

OKS showed significant differences between various postoperative time points (Figure 6A). Compared to preoperative values, a statistically significant improvement was observed at 3 months (mean difference = 16.55, 95% CI: 8.75 to 24.34, *p* = 0.0002), 6 months (mean difference = 21.64, 95% CI: 16.30 to 26.98, *p* < 0.0001), and 12 months (mean difference = 22.36, 95% CI: 17.29 to 27.43, *p* < 0.0001) following surgery. Significant improvements were also detected between 1 and 3 months (mean difference = 7.18, 95% CI: 0.85 to 13.51, *p* = 0.0227), 1 and 6 months (mean difference = 12.27, 95% CI: 4.53 to 20.02, *p* = 0.0020), and 1 and 12 months (mean difference = 13.00, 95% CI: 3.94 to 22.06, *p* = 0.0044). No other comparisons reached statistical significance, suggesting a plateau in clinical improvement after the third postoperative month.

FJS showed statistically significant improvements over time between postoperative time points (Figure 6B). A significant increase in FJS was observed between 1 and 6 months (mean difference = 19.73, 95% CI: 5.93 to 33.52, *p* = 0.0052), as well as between 1 and 12 months (mean difference = 22.64, 95% CI: 4.82 to 40.45, *p* = 0.0116). No further significant differences were found, indicating that the majority of improvement in joint awareness occurred within the first 6 months following surgery.

Since all patients achieved full extension pre- and post-operatively, ROM was evaluated and reported as the total span of flexion–extension achieved. ROM showed significant improvement over time, particularly after the first postoperative month (Figure 6C). Compared to preoperative values, statistically significant increases in ROM were observed at 3 months (mean difference = 18.18, 95% CI: 5.20 to 31.17, *p* = 0.0053), 6 months (mean difference = 19.45, 95% CI: 7.77 to 31.14, *p* = 0.0014), and 12 months (mean difference = 20.55, 95% CI: 7.61 to 33.48, *p* = 0.0020). Further significant improvements were noted when comparing 1 month with 3 months (mean difference = 11.91, 95% CI: 6.09 to 17.73, *p* = 0.0003), 6 months (mean difference = 13.18, 95% CI: 7.83 to 18.53, *p* < 0.0001), and 12 months (mean difference = 14.27, 95% CI: 7.79 to 20.75, *p* = 0.0001). The comparisons of the remaining timepoints did not show statistically significant differences, suggesting a plateau in ROM gains after the third month.

Total KSS score showed a significant improvement over time (Figure 6D). Compared to the preoperative value, statistically significant increases were observed at 3 months (mean difference = 60.36, 95% CI: 18.79 to 101.9, *p* = 0.0040), 6 months (mean difference = 78.73, 95% CI: 39.09 to 118.4, *p* = 0.0003), and 12 months (mean difference = 82.00, 95% CI: 45.83 to 118.2, *p* = 0.0001). Comparisons between 1 month and subsequent follow-ups also showed significant improvements: 3 months (mean difference = 25.91, 95% CI: 6.53 to 45.29, *p* = 0.0074), 6 months (mean difference = 44.27, 95% CI: 21.79 to 66.76, *p* = 0.0003), and 12 months (mean difference = 47.55, 95% CI: 22.71 to 72.38, *p* = 0.0004). Further significant improvements were detected between 3 and 6 months (mean difference = 18.36, 95% CI: 2.87 to 33.86, *p* = 0.0170) and between 3 and 12 months (mean difference = 21.64, 95% CI: 6.08 to 37.20, *p* = 0.0055). No statistically significant difference was found between 6 and 12 months, suggesting a plateau in functional improvement after the sixth month.

No evidence of radiolucency lines was observed on postoperative radiographs at the 3- and 12-month follow-ups in any patient.

## 4. Discussion

Residual pain after TKA remains a major concern for a non-negligible number of patients. This may be the result of a number of technical and biomechanical issues often associated with the inherent limitations of OTS implants. Based on averaged anatomical models, they may fail to accommodate the wide variability in patient morphology, potentially leading to abnormal joint kinematics, compromised joint envelope function, impaired proprioception, and reduced patient satisfaction. Implant overhang or undercoverage can lead to soft-tissue impingement, restrict normal knee motion, and provoke discomfort. In this context, PSC prostheses offer a promising alternative to address the limitations of conventional implants. However, suboptimal component alignment can result in patellar maltracking, instability, or even aseptic loosening over time. The primary aim of this study was to assess the positioning accuracy and the clinical and radiographic outcomes of knee PSCs with the aid of an inertial-based extramedullary cutting guide.

The accurate positioning of personalized knee prostheses implanted with the aid of inertial-based extramedullary cutting guides is critical in determining functional outcomes and long-term implant survival paramount. Proper component alignment in both the coronal and the sagittal planes plays a pivotal role in surgical success [20,21]. Coronal malalignment—especially excessive varus—has been linked with a higher rate of implant failure [21]. However, emerging evidence suggests that following a standardized alignment strategy for all patients might fail to achieve optimal outcomes, supporting the value of a personalized approach [20]. In the sagittal plane, improper positioning—either in excessive flexion or in extension—has been associated with complications such as increased patellofemoral joint stress, anterior knee pain, or early loosening. Specifically, sagittal femoral extension increases patellofemoral forces, potentially leading to postoperative anterior knee pain [22], whereas hyperflexion of the femoral prosthesis significantly increases the risk of early failure [21,23].

Conventional intramedullary cutting guides are prone to relevant positioning errors and are contraindicated in case of extra-articular deformities or previous hardware in the distal femur [24,25,26,27,28]. Huang et al. [28] reported that the use of conventional instruments can lead to lower limb malalignments greater than 3° in up to 27.1% of cases in the coronal plane and 43.2% of cases in the sagittal plane. Notably, tibial cuts, performed with extramedullary guides, were significantly more accurate than femoral cuts, with malalignments greater than 3° occurring in only 2.7% of cases in the coronal plane and none in the sagittal plane. In addition, Maderbacher et al. [26] found that conventional intramedullary alignment rods introduced a mean flexion error of 4.4° during distal femoral resections, confirming the limitations of standard instrumentation in controlling sagittal alignment.

In this study, in the coronal plane, both the femoral and tibial components were within 3° of the planned alignment in 100% of patients. In the sagittal plane, the femoral and tibial components were within 3° of the planned alignment in 91% and 73% of patients, respectively. Overall, the final $HKA_{CT}$ angle was within 3° of the planned personalized alignment in all patients, confirming the accuracy of the Perseus in restoring the patient’s native pre-arthritic joint line and knee geometry.

These findings are reinforced by the statistical analysis, which did not reveal any significant differences between planned and postoperative angles, suggesting that the surgical outcomes closely matched the intended alignment. The use of inertial-based extramedullary cutting guides, such as the Perseus system, offers several practical advantages that enhance its applicability across diverse clinical settings. Compared to computer-assisted surgery (CAS) and robotic systems, the Perseus guide provides a simplified and intuitive workflow, significantly reducing the learning curve required for surgeons to achieve proficiency. Unlike CAS systems, which necessitate complex setups, sensitive optical tracking equipment, and extensive surgeon training [11], the Perseus system requires only minimal additional instrumentation, with a straightforward single-pin fixation and rapid intraoperative registration process [29].

Furthermore, the literature reports that accelerometer-based navigation systems, including Perseus, do not extend surgical times, in contrast with CAS and robotic platforms, which are frequently associated with longer procedural durations due to calibration and setup requirements [11,29].

From a cost-effectiveness perspective, inertial sensor-based systems represent a highly advantageous alternative. Unlike conventional CAS systems, which entail high acquisition and maintenance costs, or robotic platforms that impose even greater financial burdens, including substantial upfront investment and recurring service contracts [30], inertial cutting guides like Perseus are inherently more economically sustainable. Accelerometer-based guides circumvent these financial constraints, as they do not depend on proprietary implants or large console infrastructures, further broadening their accessibility [11].

Importantly, this technological simplicity does not compromise the accuracy and reliability of the system. The results from the present study are consistent with other in vitro and in vivo validation studies employing inertial-based guides, which have shown precision comparable to CAS systems, a reduction in intra-subject variability, and reproducibility across different operators [12,13]. These attributes are particularly relevant for low-volume centers or facilities with limited resources, where the adoption of complex navigation or robotic systems may be unfeasible. In such settings, inertial-based guides offer an accessible pathway to enhance surgical precision and patient outcomes, even in the hands of less-experienced surgeons [12].

As for clinical and functional outcomes, our results demonstrate significant and early improvements which remained sustained throughout the follow-up period, in agreement with other works from the literature (See Supplementary Material Table S1).

Reimann et al. [31] conducted a retrospective study involving 228 patients, of whom 125 received a PSC and 103 received an OTS implant. While the ROM was comparable in both groups, the KSS, and in particular the KSS-F, achieved significantly better results in the PSC group compared to the OTS group, as well as significantly higher satisfaction. However, the authors specify that these differences should be considered with caution in light of confounding factors such as the younger age and greater expectations of the PSC group.

In a prospective study, Wendelspiess et al. [32] performed 74 PSCs and 169 OTS TKAs. Although both implants achieved significant improvements over time in PROMs, no significant differences were found between the two groups at 12-month follow-up. On the other hand, the KSS-K was significantly higher for PSC group, although patients in this group were younger and experienced less subjective impairment, potentially influencing the results.

To rule out selection bias, Schroeder et al. [33], in a single-center study, enrolled 47 patients who had previously undergone TKA with an OTS implant and prospectively underwent PSC TKA in the contralateral knee. PSCs achieved significantly higher scores for the Knee injury and Osteoarthritis Outcome Score (KOOS), Joint Replacement (JR), and FJS. Moreover, patients reported having less pain, better-perceived mobility and stability, and generally preferred the knee that underwent PSC TKA for its more natural feeling.

Vogel et al. [34] applied propensity score matching and compared 85 PSCs and 85 OTS TKAs. No differences in patient satisfaction, KOOS, FJS-12, and the EuroQol 5-Dimension 3-Level (EQ-5D-3L) were reported between the two groups. However, the EuroQol Visual Analog Scale (EQ-VAS) after 4 months and the High-Activity Arthroplasty Score (HAAS) after 1 year and 2 years were higher for PSC TKA. Also, the KSS was higher for patients with PSC compared to OTS implants at 1-year follow-up.

In a subsequent single-center, prospective cohort study, Vogel et al. [35] evaluated 51 PSCs and 51 OTS TKAs with personalized alignment applying propensity score matching. At 2 years follow-up, HAAS, KOOS, and the EQ-5D were higher for PSC TKA compared to OTS TKA. Although the satisfaction rate did not differ significantly between groups, the FJS was higher in the PSC group than in the OTS group. Furthermore, the KSS-K at the 1-year follow-up was significantly higher for the PSC group compared to the OTS group.

Studies that evaluated PSC TKA without a control group [7,36,37] also reported promising outcomes, consistent with the findings of our study.

Overall, the current literature suggests a trend towards higher KSS and FJS in patients receiving PSC compared to OTS TKA.

As for radiographical outcomes, no radiolucency lines were observed in any of the patients during the 3- and 12-month follow-up periods, indicating that the implants were well-positioned and integrated without evidence of loosening or complications. These results are consistent with previous studies that highlight the importance of accurate implant positioning for minimizing complications and enhancing survival [26,38,39,40].

Our data further support the hypothesis that a personalized, anatomically congruent approach to TKA may overcome the limitations commonly associated with standard OTS prostheses. Despite the encouraging results from our study, we acknowledge that these findings must be interpreted in consideration of important limitations. The primary limitation of this study is the small sample size (*n* = 12, that was reduced to *n* = 11 after exclusion of one patient due to software issues during surgery), which limits the statistical power and restricts the generalizability of the findings. Additionally, the absence of a control group, such as patients treated with conventional, CAS, or robot-assisted procedures with OTS implants, prevents any direct comparison and hinders the assessment of the relative advantages or potential superiority of the proposed approach. Another relevant limitation is represented by the short follow-up period, which does not allow for a comprehensive assessment of long-term (>12 months) clinical and radiological outcomes, including the evaluation of implant stability, complication, and revision rates. Such shortcomings will need to be addressed by future studies involving larger and more heterogeneous patient populations, longer follow-up periods, and appropriate control groups to robustly assess the long-term effectiveness and comparative advantages of this personalized approach.

## 5. Conclusions

The study investigated the accuracy of an inertial-based extramedullary cutting guide (Perseus) in the positioning of PSCs in TKA and evaluated the clinical and radiographic outcomes resulting from this combination. The protheses used (YourKnee^TM^) were individually designed to match the patient’s unique anatomy. In this study, the use of the Perseus inertial-based extramedullary cutting guide and 3D-printed PSI ensured precise bone preparation and accurate implant placement in accordance with personalized preoperative planning. The combination of PSCs with inertial cutting guides led to statistically significant improvements in clinical and functional outcomes up to 12 months postoperatively. Despite the small sample size, the absence of a control group, and the short-term outcomes, this integrated approach represents a viable alternative to conventional instrumentation and OTS designs, with the potential to reduce residual pain, improve early function, and improve long-term patient satisfaction. Further works will provide clinical outcomes on the basis of a longer follow-up.

## Figures and Tables

**Table 1:** Comparison of planned and post-operative alignment angles.

	Planned Mean (SD)	Post-Op Mean (SD)	Abs Error Mean (SD)	% <3°	B-A Bias (SD)	B-A 95% LoA
MDFA (°)	90.18 (1.66)	90.82 (2.10)	1.87 (0.87)	100%	−0.63 (2.04)	[−4.64,3.37]
MPTA (°)	89.27 (1.79)	89.03 (1.96)	1.67 (0.75)	100%	0.24 (1.89)	[−3.46, 3.95]
Femur F/E (°)	96.73 (3.77)	97.68 (4.79)	1.89 (1.24)	91%	−0.95 (2.11)	[−5.09, 3.19]
Tibial slope (°)	94.55 (1.27)	95.4 (3.46)	2.45 (0.87)	73%	−0.85 (2.56)	[−5.86, 4.16]
HKA_CT_ (°)	−0.55 (1.57)	−0.16 (−0.27)	1.36 (1.06)	100%	−0.39 (1.73)	[−3.79, 3.01]

Abbreviations: abs: Absolute, B-A: Bland–Altman, F/E: Flexion/Extension, $HKA_{CT}$: CT-derived Hip Knee Angle, LoA: Limits of Agreement, MDFA: Medial Distal Femoral Angle, MPTA: Media Proximal Tibial Angle, SD: Standard Deviation.

**Table 2:** Patient reported outcomes and knee range of motion from pre-operative baseline to 1-, 3-, 6-, and 12-month follow-up.

	PRE Mean (SD)	FU 1 Mean (SD)	FU 3 Mean (SD)	FU 6 Mean (SD)	FU 12 Mean (SD)	*p*-Value
OKS (score)	20.64 (2.77)	30 (9.54)	37.18 (6.90)	42.27 (4.34)	43 (4.12)	*p* < 0.0001
ROM (°)	90.91 (11.14)	97.18 (9.12)	109.09 (9.07)	110.36 (8.44)	111.45 (8.21)	*p* < 0.0001
KSS f (score)	46.09 (11.54)	59.55 (17.81)	74.09 (13.75)	85.00 (12.45)	86.82 (10.07)	*p* < 0.0001
KSS k (score)	44.55 (11.28)	65.55 (12.72)	76.91 (12.16)	84.36 (11.86)	85.82 (9.73)	*p* < 0.0001
KSS tot (score)	90.64 (17.25)	125.09 (30.19)	151.00 (25.57)	169.36 (23.57)	172.64 (19.46)	*p* < 0.0001
FJS (score)		65.34 (19.24)	75.39 (25.65)	85.09 (17.14)	87.97 (15.28)	*p* = 0.0031

Abbreviations: FJS: Forgotten Joint Score, FU: Follow-Up, KSS: Knee Society Score, OKS: Oxford Knee Score, PRE: Preoperative, ROM: Range of Motion.

## Data Availability

The data that support the findings of this study are available from the corresponding author upon request.

## Supplementary Materials

The following supporting information can be downloaded at: https://www.mdpi.com/article/10.3390/medicina61091554/s1, Figure S1: Quantile-Quantile plots to assess normality of residuals of OKS, FJS, ROM, and KSS.; Table S1: Literature review of clinical and functional outcomes after PSC (and OTS, in case a control group was present) TKA.

## Abbreviations

The following abbreviations are used in this manuscript:

AP	Antero-Posterior
ASA	American Society of Anesthesiologists
BMI	Body Mass Index
CR	Cruciate Retaining
CT	Computed Tomography
EQ-5D-3L/EQ-VAS	EuroQol scores
FJS	Forgotten Joint Score
FLRs	Full Leg Radiographs
FU	Follow-Up
HAAS	High-Activity Arthroplasty Score
$HKA_{CT}$	CT-derived Hip–Knee–Ankle
JR	Joint Replacement
KL	Kellgren–Lawrence
KOOS	Knee Injury and Osteoarthritis Outcome Score
KSS	Knee Society Score
MA	Mechanical Alignment
MDFA	Medial Distal Femoral Angle
ML	Medio-Lateral
MPTA	Medial Proximal Tibial Angles
OA	Osteoarthritis
OKS	Oxford Knee Score
OTS	Off-the-shelf
PRE	Pre-operative
PROMs	Patient Reported Outcomes Measures
PSC	Patient Specific Components
PSI	Patient Specific Instrument
ROM	Range of Motion
SD	Standard Deviation
TKA	Total Knee Arthroplasty
VAS	Visual Analogue Scale
