# Supplementary material for: Accuracy and Early Outcomes of Patient-Specific TKA Using Inertial-Based Cutting Guides: A Pilot Study
Source: Medicina (Kaunas). 2025 Aug 29;61(9):1554. doi: 10.3390/medicina61091554 (PMC12471548; doi:10.3390/medicina61091554)
Supplement: Supplementary file 1 [file medicina-61-01554-s001.zip › medicina-3806011-supplementary.pdf]

Supplementary material

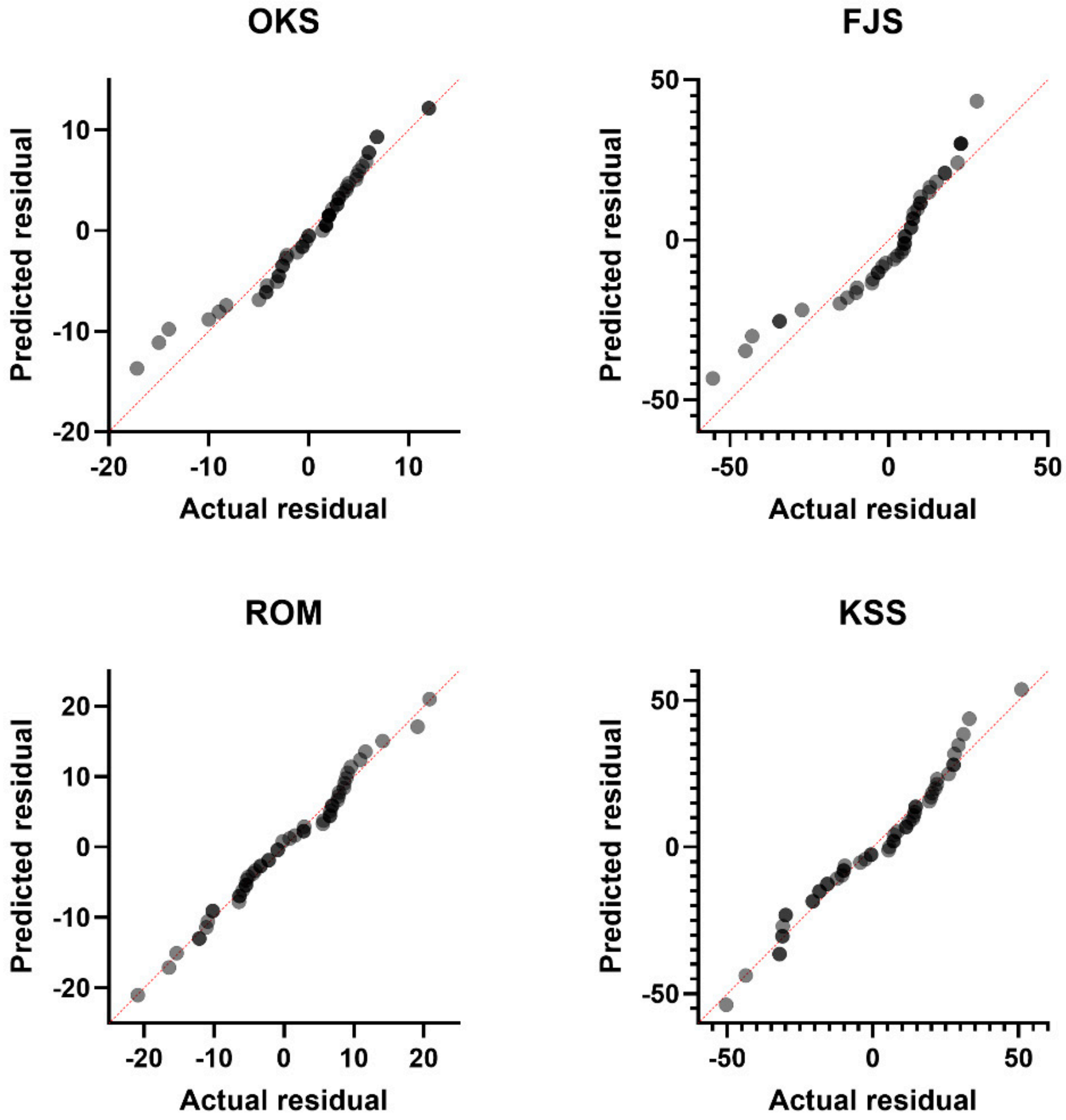

Figure S1: Quantile-Quantile plots to assess normality of residuals of OKS, FJS, ROM, and KSS.

Table S1. Literature review of clinical and functional outcomes after PSC (and OTS, in case a control group was present) TKA.

Author, year	Implant	FU	OKS	FJS	ROM	KSSf	KSSk
This study	11 PSC	12 m	43.0 ± 4.1	88.0 ± 15.3	111.5 ± 8.2	86.8 ± 10.1	85.8 ± 9.7
Reimann 2019 [28]	125 PSC	2-3 y	-	-	110 ± 15.0	82.0 ± 19.2	82.4 ± 13.1
	103 OTS		-	-	110 ± 13.8	68.0 ± 18.7	78.3 ± 13.8
Wendelspiess 2022 [29]	74 PSC	12 m	-	-	-	-	94.2 ± 7.5
	169 OTS		-	-	-	-	88.9 ± 8.6
Schroeder 2022 [30]	47 PSC	2.3 y	-	67.9 ± 26.4	-	-	-
	47 OTS	6.7 y	-	58.3 ± 29.2	-	-	-

Vogel 2023 [31]	85 PSC	12 m	-	65.0 ± 25.5	-		94.6 ± 6.1
	85 OTS		-	65.4 ± 26.4	-	-	89.0 ± 8.0
Vogel 2024 [32]	51 PSC	2 y	-	77 ± 23	-	-	94 ± 4
	51 OTS		-	67 ± 28	-	-	87 ± 8
Moret 2021 [6]	25 PSC	12 m	-	73 ± 19.7	129 ± 5.3	-	94 ± 5.8
Ratano 2022 [33]	227 PSC	12 m	-	-	-	96.2 ±9.1	94.2 ± 9.1
Gousopoulos 2023 [34]	140 PSC	33.5 m	39.6±7.6	69.0±27.7	-	-	-

Abbreviation: FJS: Forgotten Joint Score, **FU**: Follow-Up, **KSS_{f-k}**: Knee Society Score Functional/Knee, **OTS:** Off-The-Shelf, **OKS**: Oxford Knee Score, **PSC:** Patient-Specific Components, **ROM**: Range Of Motion.
